# Generation and Application of the Zebrafish *heg1* Mutant as a Cardiovascular Disease Model

**DOI:** 10.3390/biom10111542

**Published:** 2020-11-12

**Authors:** Shuxian Lu, Mengyan Hu, Zhihao Wang, Hongkai Liu, Yao Kou, Zhaojie Lyu, Jing Tian

**Affiliations:** Key Laboratory of Resource Biology and Biotechnology in Western China, Ministry of Education, School of Medicine, Northwest University, Xi’an 710069, China; lushuxian@stumail.nwu.edu.cn (S.L.); humengyan@stumail.nwu.edu.cn (M.H.); zhihaowang9264@gmail.com (Z.W.); liuhongkai@stumail.nwu.edu.cn (H.L.); kouyao@stumail.nwu.edu.cn (Y.K.); lyuzhaojie@stumail.nwu.edu.cn (Z.L.)

**Keywords:** cardiovascular disease (CVD), dilated cardiomyopathy (DCM), thrombosis, zebrafish model, CRISPR/Cas9, *heg1*, Traditional Chinese Medicine (TCM), drug screening

## Abstract

Cardiovascular disease (CVD) is the leading cause of global mortality, which has caused a huge burden on the quality of human life. Therefore, experimental animal models of CVD have become essential tools for analyzing the pathogenesis, developing drug screening, and testing potential therapeutic strategies. In recent decades, zebrafish has entered the field of CVD as an important model organism. HEG1, a heart development protein with EGF like domains 1, plays important roles in the development of vertebrate cardiovascular system. Loss of HEG1 will affect the stabilization of vascular endothelial cell connection and eventually lead to dilated cardiomyopathy (DCM). Here, we generated a *heg1*-specific knockout zebrafish line using CRISPR/Cas9 technology. Zebrafish *heg1* mutant demonstrated severe cardiovascular malformations, including atrial ventricular enlargement, heart rate slowing, venous thrombosis and slow blood flow, which were similar to human heart failure and thrombosis phenotype. In addition, the expression of zebrafish cardiac and vascular markers was abnormal in *heg1* mutants. In order to apply zebrafish *heg1* mutant in cardiovascular drug screening, four Traditional Chinese Medicine (TCM) herbs and three Chinese herbal monomers were used to treat *heg1* mutant. The pericardial area, the distance between sinus venosus and bulbus arteriosus (SV-BA), heart rate, red blood cells (RBCs) accumulation in posterior cardinal vein (PCV), and blood circulation in the tail vein were measured to evaluate the therapeutic effects of those drugs on DCM and thrombosis. Here, a new zebrafish model of DCM and thrombosis was established, which was verified to be suitable for drug screening of cardiovascular diseases. It provided an alternative method for traditional in vitro screening, and produced potential clinical related drugs in a rapid and cost-effective way.

## 1. Introduction

The cardiovascular system is the first organ system to form and function in vertebrate embryos. Cardiovascular disease (CVD) generally refers to the heart ischemic or hemorrhagic diseases caused by hyperlipidemia, blood viscosity, atherosclerosis, and hypertension, etc. [1]. An article published in *The Lancet* in 2020 investigated cardiovascular disease admissions and deaths among middle-aged adults in 21 countries. The results showed that cardiovascular disease was the most common cause of hospitalization (23.9%) and the most common cause of death (40%) [2]. Therefore, the development of effective and safe therapeutic drugs for CVD has proven to be challenging.

**Figure 1:**
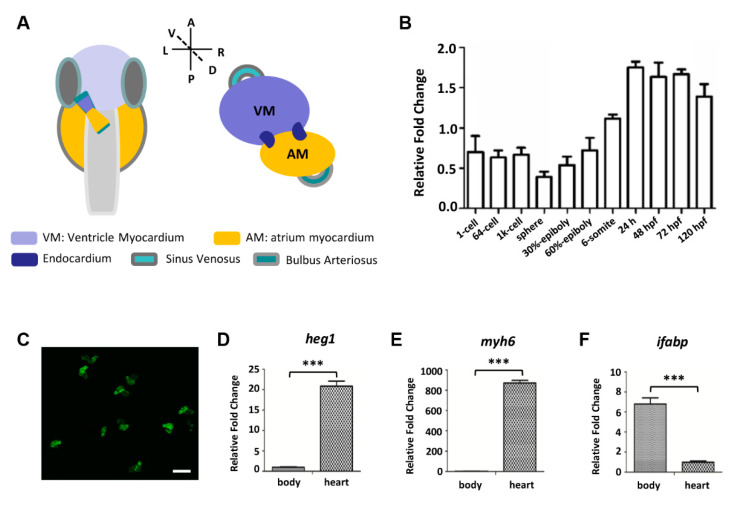
The expression level of *heg1* in zebrafish heart tissue. (**A**) Overview of zebrafish embryos heart, dorsal view (VM: Ventricle Myocardium; AM: atrium myocardium). (**B**) qRT-PCR analysis of *heg1* transcript levels from 1-cell to 120 hpf. (**C**) Purified heart tissues from *Tg(cmlc2:eGFP)* transgenic fish embryos at 72 hpf. *heg1* (**D**) and *myh6* (**E**) were highly expressed in purified heart tissue compared with the expression in body tissue as determined by qRT-PCR. (**F**) The expression level of *ifabp* in purified heart tissue was much lower than that in body tissue as analyzed by qRT-PCR. Scale bar: 100 μm. *** indicates *p* < 0.001 by Student’s *t*-test.

Zebrafish, a useful tool to study human diseases, is an excellent model for cardiovascular disease research. Zebrafish embryonic heart has two chambers, atrium and ventricle. Heart development in zebrafish starts very early; cardiac progenitors can be found at the blastula stage, about 5 h after fertilization (hpf). At 24 hpf, the heart tube forms, and leads to the ventricle to be anterior and the atrium to be posterior. The coordinated atrial and ventricular contraction develops at 36 hpf (Figure 1A) [3]. Venous blood flows into atrium then into the ventricle through the one-way atrioventricular valve. The ventricle then pumps blood to the body through a one-way outflow valve. The general pattern of the zebrafish heart formation is similar to that of early mammalians. Therefore, the heart of zebrafish at 48 hpf is comparable in morphology and physiology to the heart of a 12-day mouse or 35-day human embryo [4]. Zebrafish hearts have coronary blood vessels, and their heart rate (HR) (120/140 beats per minute) is closer to humans (60/100 beats per minute) than mice (300/600 beats per minute) [5]. One notable exception to zebrafish blood circulation is that, during the first week after fertilization, embryos do not rely on convective circulation, because they obtain sufficient oxygen through diffusion. This allows the embryos with cardiovascular defects to continue to develop until they outgrow the diffusion distance of oxygen. This facilitates more extensive phenotypic assessment of mutations, which in mammals can lead to very early embryonic lethality [6,7]. In addition, zebrafish thrombocytes are homologous to mammalian platelets, and the hemostatic mechanisms of zebrafish cells and body fluids are similar to those of human beings, which indicates that zebrafish can be used as a model for hemostasis and thrombosis in mammals [8,9]. Moreover, zebrafish transparent embryos make it easy to observe phenotypic changes, which make zebrafish a rapid, simple and low-cost cardiovascular drug-screening system [10].

Dilated cardiomyopathy (DCM) model and thrombosis model are two of the most popular zebrafish models that are widely used to mimic human CVD. For example, phenylhydrazine (PHZ) [11], photochemical excitation [12], and arachidonic acid (AA) [13] induced thrombosis models are used to evaluate antithrombotic drugs, and verapamil-induced heart failure model is used to assess the therapeutic effects of drug treatment. However, drug or physical injury modeling is a complex process, time-consuming, and characterized by phenotypic instability. Here, we hope to construct a stable zebrafish mutant which can be used for cardiovascular drug screening.

To date, there are more than 300 genes known to be related to congenital heart disease (CHD) [14]. The heart development protein with EGF-like domains 1 (HEG1) receptor is selectively expressed in endothelial cells and endocardium cells. During embryonic development, heg1, as an important intercellular adhesion cadherin protein, maintains the function and integrity of heart and blood vessels. In 2003, Mably et al. [15] first identified the *heg1* gene through a zebrafish N-ethyl-N-nitrosourea (ENU) mutagenesis screening. Zebrafish *heg1* mutants show cardiac valve defect, heart swelling, and delayed blood flow. In mice with *Heg1* gene mutation, the vascular endothelial barrier is weakened, resulting in cardiovascular diseases such as sepsis, atherosclerosis, and multiple sclerosis [15,16,17].

Due to the advantages of the zebrafish system, it is gaining popularity, especially in studying the molecular mechanism of human diseases and related drug screening. Using genome editing technology, zebrafish strains can be created with specific pathogenic genes, which can mimic some of the disease gene mutations found in humans. The construction of zebrafish mutants and their application in drug screening have been reported. For example, Duchenne muscular dystrophy (DMD) is the most common and severe form of muscular dystrophy, which is caused by mutations in the Dystrophin (DMD) gene [18]. Zebrafish DMD mutants *sapje* and *sapje-like* exhibited dystrophic features of skeletal muscle at 72 hpf, which are ideal models for studying DMD [19]. Potential therapeutic drugs’ screening carried in these DMD mutants identified at least seven chemicals that could rescue muscle phenotype found in the dystrophin-null fish [19]. Nav1.1 (SCN1A) mutation is one of the main causes of Dravet syndrome. Zebrafish *scn1a* mutants exhibited spontaneous abnormal electrographic activity, hyperactivity and convulsive behaviors, so they can simulate the clinical phenotype of Dravet syndrome patients. A large-scale drug screening based on 320 compounds identified that clemizole significantly inhibited convulsive behavior and electrographic seizures of mutant embryos, which confirmed the potential clinical significance of clemizole in the treatment of Dravet syndrome [20]. This represents that zebrafish mutants can be used to identify novel therapeutic drugs for a rapid and cost-effective purpose.

In this study, we generated the zebrafish *heg1* mutants by CRISPR/Cas9 technology. Zebrafish *heg1* mutant embryos developed pericardial swelling, pericardial edema, and decreased heart rate, which were similar to the pathophysiology characteristics observed in heart failure patients [14]. *heg1* mutants’ embryos also exhibited venous congestion, reduced cardiac output and blood flow velocity, which was consistent with the features of zebrafish thrombosis model. To assess the zebrafish *heg1* mutant in cardiovascular drug screening, four Traditional Chinese Medicine (TCM) herbs (*Salviae miltiorrhiza* (Danshen), *Radix astragali* (Huangqi), *Hirudo* (Shuizhi) and *Myrrha* (Moyao)) and three Chinese herbal monomers (salvianolic acid B, paeoniflorin, ferulic acid) were used to treat *heg1* mutant. Here, we provided a new zebrafish model of DCM and thrombosis, which could be applied for potential clinical CVD drug screening.

## 2. Materials and Methods

### 2.1. Zebrafish Care and Maintenance

All adult zebrafish were housed in aquaculture facility with a standard light cycle (14 h light, 10 h dark) [21]. Embryos were obtained by natural crosses of wild-type (wt) or transgenic fish [22]. The following lines were used: AB strain, *Tg*(*cmlc2:eGFP*) [23], and *Tg*(*flk1:eGFP*) [24]. All experimental procedures on zebrafish were approved by the Institutional Animal Care and Use Committee of Northwest University and carried out in accordance with the approved guidelines (NWU-AWC-20180601Z).

### 2.2. Purification of Hearts from Zebrafish Embryos

Hearts were extracted from *Tg*(*cmlc2: eGFP*) embryos according to the method adapted from Burns [25]. A total of 200 to 300 embryos at 72 hpf were placed into a 1.5 mL tube. Excess water was removed and 1 mL of L-15 medium containing 10% of fetal calf serum was added. A 5 mL syringe with a 20-gauge needle was used to destroy the pericardium and to release cardiac tissues by repeatedly passing embryos through syringe about 15 times. Fragmented embryos were placed in a 100mm dish with cold L-15 medium; hearts were collected with a glass pipette under fluorescence microscope and placed in cold L-15 medium. After centrifugation at 4 °C 1000 g for 15 min, the supernatant was discarded and the precipitated cardiac tissue was collected for further study.

### 2.3. Generation of heg1 Mutant Zebrafish

The procedure for CRISPR/Cas9 editing in zebrafish was described in detail in [26,27]. Zebrafish *heg1* target sites were designed using the website https://www.crisprscan.org/?page=sequence [28] (2015–2019, Giraldez lab, New Haven, CT, USA). The provided sites were then screened in NCBI. *heg1* guide RNA (gRNA) (5′- CCGTGTGCTTTTCACGGCCGC -3′) that specifically targeted exon 1 in the zebrafish genome were chosen for the interruption of the *heg1* gene. The gRNA was transcribed in vitro using the MAXIscript T7 kit (Ambion, Waltham, MA, USA). The mixtures including 100 pg gRNA and 0.5pmol Cas9 protein (NEB, Ipswich, UK) were injected into one-cell stage embryos using PLI-100A Plus Pico Injector (Digitimer North America, Lauderdale, FL, USA). More than a dozen embryos were lysed and sequenced to examine gRNA efficiency, and the remaining embryos were raised to adulthood to obtain the mosaic founders. Mosaic zebrafish were crossed with AB wild-type zebrafish to obtain *heg1* heterozygotes and genotyped by Sanger sequencing. *heg1^+/−^* were further crossed with the cardiovascular system specific transgenic reporter lines *Tg*(*cmlc2:EGFP*) or *Tg*(*flk1:EGFP*) for phenotypic analysis. Genotyping primers of *heg1* are summarized in Supplemental Table S1.

### 2.4. Cardiac Phenotype Analysis

Embryos were collected at 48, 72, and 96 hpf for phenotype analysis. Zebrafish embryos were mounted in 5% methylcellulose. Images were captured using a stereomicroscope (SMZ25, Nikon, Tokyo, Japan) for morphology observation. Photographs were quantified using NIS-Elements BR software (Ver5.30.00, Nikon, Tokyo, Japan) to determine the pericardial area and sinus venosus and bulbus arteriosus (SV-BA) distance per field of view.

### 2.5. Blood Flow Rate and Heart Rate Statistics

Video imaging of the red blood (RBCs) movement in the posterior cardinal vein (PVC) for 96 hpf embryos was captured by stereomicroscope (SMZ25, Nikon, Tokyo, Japan). Using DanioScope software (DVOC-0041, Noldus, Wageningen Netherlands) to analyze the movement ratio of RBCs based on changes in pixel density, the result was expressed as a ratio of blood flow pixel value to surrounding tissue. The heart rate of embryos per unit of time was counted with a stopwatch and a counter, expressed as beats per minute (BPM).

### 2.6. Real-Time Quantitative PCR (qRT-PCR) Analysis

Total RNA was extracted from fresh zebrafish embryo tissues using TRIzol reagent (Ambion, Austin, TX, USA) as described in [29] with modifications. cDNA synthesis was performed using SuperScriptTM reverse transcriptase system (Invitrogen, Carlsbad, CA, USA). qRT-PCR was performed with SYBR Green PCR Master Mix (Kapa Biosystems, Boston, MA, USA) and ViiA 7 Real-Time PCR System (ThermoFisher, Waltham, MA, USA). Primer sequences are listed in Supplementary Table S1.

### 2.7. Whole-Mount in situ Hybridization and Red Blood Cell Staining Assay

Digoxigenin (DIG)-labeled riboprobes of *cmlc1* were synthesized using RNA Labeling Kit (Roche, Basel, Switzerland). Whole-mount in situ hybridization (WISH) assays were performed as described previously [29,30]. Briefly, zebrafish embryos at different stages were collected and fixed in 4% paraformaldehyde at 4 °C overnight. After a 4-h pre-hybridization incubation at 65 °C, embryos were subsequently incubated with the antisense probes overnight. Anti-digoxigenin antibody (Roche, Basel, Switzerland) was used to bind the probes overnight at 4 °C. The embryos were stained using BM purple (Roche, Basel, Switzerland), and photographed under Nikon SMZ25 microscope system.

The 4 dpf embryos were fixed in 4% paraformaldehyde (PFA) for four hours at room temperature, then red blood cells (RBC) were stained by o-dianisidine solution comprised of 2.9 mM 3,3′-Dimethoxybenzidine, 0.1 M NaOAc, 30% H_2_O_2_, and 50% absolute ethanol in the dark for 30 min. Stained embryos were washed with PBST three times and stored in 80% glycerol for imaging using Nikon SMZ25 Microscope.

### 2.8. Drug Preparation

The TCM herbs (*Salviae miltiorrhiza* (Danshen), *Radix astragali* (Huangqi), *Hirudo* (Shuizhi) and *Myrrha* (Moyao)) were provided from Buchang Pharmaceuticals (Shaanxi, China). The *Salviae miltiorrhiza*, *Radix astragali* and *Hirudo* were macerated in 50 mL distilled water for 30 min, and were ultrasonicated (250 W, 50 kHz) for 30 min, evaporated to dry powder and dissolved in H_2_O to a final concentration 1 g/mL. *Myrrha* was prepared by alcohol extraction and dissolved in DMSO. The stock concentrations of four herbs were 1 g/mL.

Salvianolic acid B (SS8100), paeoniflorin (SP8030), ferulic Acid (SF8030) and aspirin (IA0550) were purchased from Solarbio Company (Beijing, China). All monomers were dissolved in DMSO at stock concentration of 10 mM.

### 2.9. Assessment Effects of Drugs on heg1 Mutant

*heg1* mutants embryos at 48 hpf were collected and placed into 6-well plates. To optimize the concentrations of TCM herbs and monomers, the following doses were used: *Radix astragali* (1100, 1300, 1600, 2000, and 2200 mg/L), *Salviae miltiorrhizae* (1800, 2000, 2200, 3000, and 3800 mg/L), *Hirudo* (300, 400, 500, 1000, and 2000 mg/L), *Myrrha* (50, 100, 150, 300, and 400 mg/L), salvianolic acid B (5, 10, 15, 20, 30 μM), paeoniflorin (5, 10, 15, 20, 30 μM), ferulic acid (5, 10, 15, 20, 30 μM), and aspirin (60, 80, 100, 120, 140 μM) for 2 days, respectively. The recovery rate of venous thrombosis was recorded at the end of the treatment; 40 embryos per group were used for statistics analysis.

The optimal concentrations of *Radix astragali* 1300 mg/L, *Salviae miltiorrhiza* 2000 mg/L, *Hirudo* 400 mg/L, and *Myrrha* 100 mg/L, salvianolic acid B 20 μM, paeoniflorin 15 μM, ferulic acid 5 μM and aspirin 120 μM were treated on *heg1* mutant embryos at 48 hpf for 2 days, respectively. Control groups were treated with 0.1% DMSO or 0.9% sodium chloride to confirm that the solvent did not have an adverse effect on embryos. After treatment, ten embryos from each group were randomly selected for phenotype observation, cardiac morphology measurement, blood flow rate and heartbeat statistics’ analysis.

### 2.10. Statistical Analysis

All experiments were repeated at least three times. Data analysis was performed by using SPSS Statistical Package version 17 (IBM, Armonk, NY, USA). Student’s *t*-test was applied for comparisons among different groups. All data are presented as mean ± SE, and *p* < 0.05 was considered significant statistically.

## 3. Results

### 3.1. Zebrafish heg1 was Highly Expressed in Cardiac Tissue

Levels of *heg1* transcripts were analyzed by qRT-PCR. As shown in Figure 1B, the maternal mRNA signals of *heg1* could be detected during the cleavage (1-cell, 64-cell). The zygotic *heg1* was expressed through the blastula stage (1k-cell, sphere), gastrulation (30%-epiboly, 60%-epiboly), somitogenesis (6-somite), reached the highest level at 24 hpf, and maintained high expression until 120 hpf, as detected (Figure 1B). To further investigate the tissue-specific expression of *heg1*, zebrafish embryonic hearts were dissected and collected at 72 hpf (Figure 1C). The expression of *heg1* in heart was twenty times higher than that in body tissues (Figure 1D). As tissue-specific markers, *myosin heavy chain 6* (*myh6*) is highly expressed in atrium [31], and fatty acid binding protein 2 (*fabp2/ifabp*) is known to be specifically expressed in intestinal epithelium [32]. As shown in Figure 1E,F, the expression of *myh6* in zebrafish cardiac tissue was more than 800 times higher than that in other tissues, while the expression level of ifabp in purified cardiac tissues was very low (Figure 1E,F). The results of quantitative analysis of gene expressions revealed that *heg1* gene was much more abundant in heart tissue than in other tissues, which strongly indicated that *heg1* played an important role in cardiac development.

### 3.2. heg1 Deficient Zebrafish Was Generated Using CRISPR/Cas9 Technology

**Figure 2:**
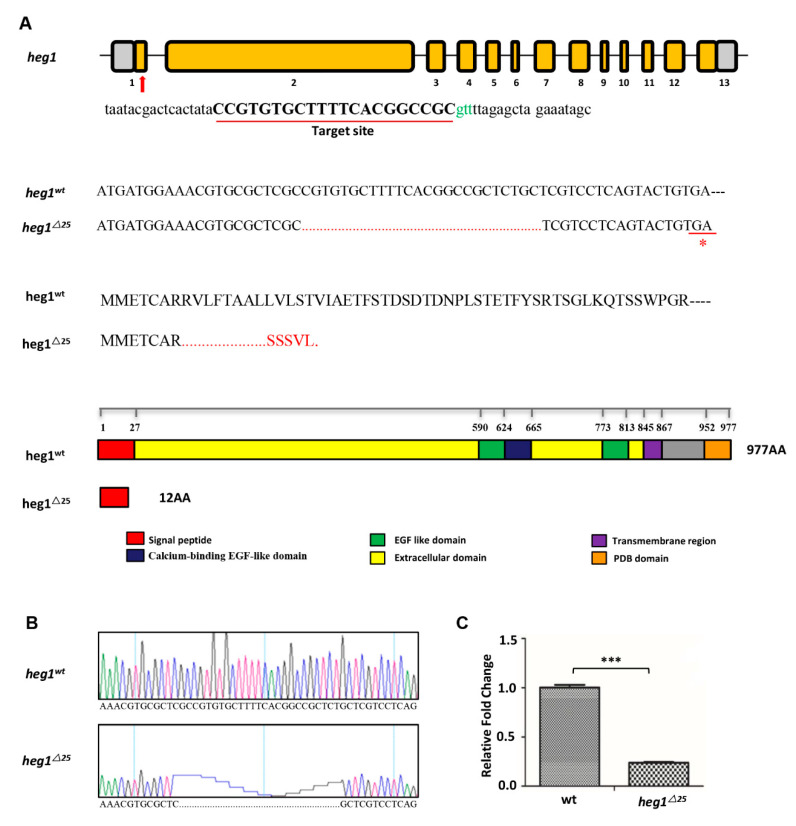
The generation of *heg1* deficient zebrafish using CRISPR/Cas9 technology. (**A**) Schematic view of zebrafish heg1 genomic and protein domains. The gRNA sequence for the exon 1 targeting site was labelled by a red line. The CRISPR/Cas9-induced mutation (25-base deletion) in *heg1* exon1 and a truncated 12 amino acids at the N-terminus were shown. (**B**) Sanger sequencing confirmed the 25-nt deletion mutation. (**C**) qRT-PCR confirmation that *heg1* expression was significantly decreased in *heg1^∆^^25^* embryos, *n* = 30 embryos per group. Data are represented as mean ± SE. *** *p* < 0.001.

Zebrafish *heg1* gene is located on chromosome 9 and consists of 13 exons and 12 introns (Figure 2A). *heg1* gRNA comprising a 21-base sequence was designed for the gene-specific (Figure 2A). We were able to generate a 25-nt deletion in exon1, which led to a frameshift and a premature stop codon (TGA), resulting in a truncated protein with only 12 amino acids left (Figure 2A,B). The mutation disrupted all known functional domains of heg1 protein. qRT-PCR analysis editing of exon 1 showed that the transcript of *heg1* mRNA was significantly eliminated in *heg1^∆25^* embryos (Figure 2C), which might be due to the effect of nonsense-mediated decay (NMD) [32]. Thus, it indicated that *heg1^∆25^* was a loss-of-function mutation.

### 3.3. Abnormal Cardiac Formation in heg1^∆25^ Mutants

**Figure 3:**
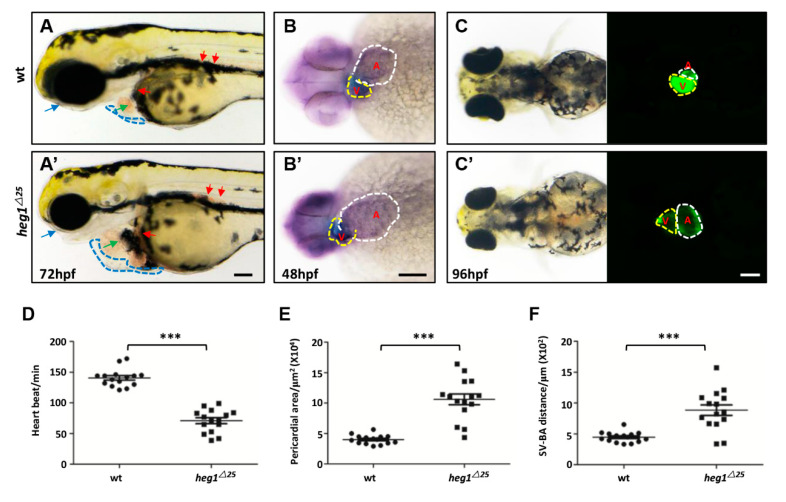
*heg1* deficiency leaded to abnormal cardiac development in zebrafish embryos. (**A**,**A’**) Lateral view of zebrafish larvae at 72 hpf. Representative images of the *heg1^∆^^25^* and wt embryos, exhibiting pericardial edema (blue dotted-line boxes), enlarged heart size (green arrow), venous congestion (red arrow), and eye edema (blue arrow). (**B**,**B’**) Representative images of the *heg1^∆^^25^* and wt embryos at 48 hpf stained for the heart marker *cmlc1*. Note the enlargement heart in *heg1^∆^^25^* mutants (V: Ventricular, yellow dotted-line boxes; A: atria, white dotted-line boxes, ventral view). (**C**,**C’**) The heart morphology was delineated by *Tg*(*cmlc2:GFP*) (ventral view). (**D**) Heart rate in wt and *heg1^∆25^* mutant zebrafish larvae (*n* = 15 embryos/group). (**E**) The pericardial area in wt and *heg1^∆^^25^* mutant zebrafish larvae (*n* = 15 embryos/group). (**F**) The SV-BA distance in wt and *heg1^∆^^25^* mutant zebrafish larvae (*n* = 15 embryos /group). Scale bar: 100 μm. *** indicates *p* < 0.001 by Student’s *t*-test.

Obvious cardiovascular malformation of *heg1^∆^^25^* mutants were observed from 48 hpf. The mutants exhibited progressively severe edema, first in the pericardial sac and then around the eyes and yolk sac (Figure 3A,A’). The heart wall of the *heg1^∆^^25^* mutant is grossly distended, and congestion was seen both in the heart and in the posterior cardinal vein (Figure 3A,A’). Cmlc (cardiac myosin light chain) family specifically encodes myosin and is expressed in the heart during the development of the cardiovascular system [33]. The WISH of *cmlc1* (Figure 3B,B’) and *bmp4* (Figure S1B,B’) showed enlarged heart size in *heg1^∆^^25^* mutant with overtly expanded atrium. To better analyze the cardiac defects in *heg1^∆^^25^* mutant, *heg1^∆^^25^* was crossed with the cardiomyocyte specific transgenic reporter line *Tg*(*cmlc2:eGFP*) to produce the bi-genic line (*heg1^∆^^25^;cmlc2:eGFP*). This (*heg1^∆^^25^;cmlc2:eGFP*) line also showed enlargement of heart chambers (Figure 3C,C’).

Under normal circumstances, the heart is beating spontaneously and regularly. The change in heart rhythm or interference may be the cause of cardiac malfunction. In *heg1^∆^^25^* mutant embryos, heart rate was markedly reduced compared to wt embryos (Figure 3D). Meanwhile, the cardiac area in the *heg1^∆^^25^* mutant was almost double that of the wt embryos (Figure 3E), reflecting severe heart edema and pericardial effusion. The distance between sinus venosus and bulbus arteriosus (SV-BA) is an indicator to measure the degree of cardiac looping. *heg1^∆^^25^* mutant exhibited a significant prolongation of the SV-BA distance (Figure 3F). The zebrafish *heg1^∆25^* mutant cardiac defects was resembled to human heart failure (HF) caused by dilated cardiomyopathy (DCM).

### 3.4. Abnormal Vascular Development and Blood Stagnation in heg1^∆25^ Mutants

**Figure 4:**
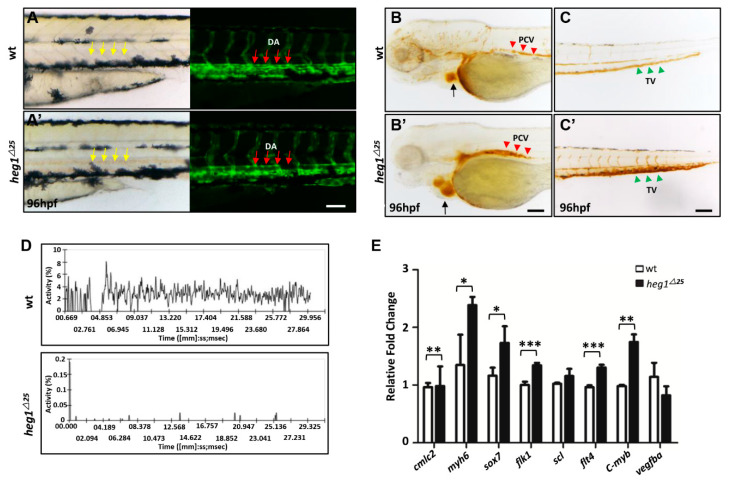
*heg1* deficiency leaded to poor blood flow and abnormal vascular development in zebrafish embryos. (**A**) Lateral view of zebrafish larvae at 96 hpf. Representative images of the *heg1^∆^^25^* and wt embryos, exhibiting blood congestion (yellow arrows), and dilation of dorsal aorta (DA) lumen (red arrows) in caudal vein. (**B**,**C**) Representative images of the *heg1^∆^^25^* and wt embryos, showing red blood cells (RBCs) accumulation in posterior cardinal vein (PCV) (red arrowhead), tail vein (TV) (green arrowhead) and heart (black arrow). (**D**) The movement ratio of RBCs based on changes in pixel density of PVC. (**E**) The expressions of cardiovascular markers, as determined by qRT-PCR, were significantly changed in *heg1^∆25^* mutants at 48 hpf. Data are represented as mean ± SE from three independent experiments, * *p* < 0.05, ** *p* < 0.01, and *** *p* < 0.001 (Student’s *t*-test).

Cardiac malformations are usually accompanied by vascular dysplasia and poor blood circulation. Embryonic endothelial cells can be easily observed in zebrafish using transgenic fluorescent zebrafish *Tg*(*flk1:eGFP*) line. *heg1^∆^^25^* was crossed with *Tg*(*flk1:eGFP*) to produce the bi-genic line (*heg1^∆^^25^*; *flk1:eGFP*) for vascular malformation observation. In *heg1^∆^^25^* mutant embryos, blood often accumulated and coagulated in heart chambers or blood vessels (Figure 3A,A’ and Figure 4A,A’). Fluorescence microscope images further revealed the dilation of dorsal aorta (DA) lumen in *heg1^∆25^* embryos at 96 hpf (Figure 4A,A’). Red blood cells (RBCs) staining in *heg1^∆^^25^* embryos at 96 hpf showed the accumulation of RBCs not only in the heart, but also in the posterior cardinal vein (PCV) and tail vein (TV) (Figure 4B,B’,C,C’). The movement of the RBCs in the tail vein of 96 hpf embryos was validated by using DanioScope software. It could be clearly seen that the relative movement of RBCs in *heg1^∆^^25^* embryos was almost abolished (Figure 4D); qRT-PCR results also confirmed that thrombotic markers, such as *f2* and *tbxasl*, were also abnormally increased in *heg1^∆^^25^* mutants (Figure S1A).

Furthermore, the expressions of cardiovascular markers were significantly changed as detected by qRT-PCR analysis in *heg1^∆25^* mutant (Figure 3E). As shown in Figure 4E, the expressions of *cmlc2* and *myh6*, specific markers for heart tissue, were abnormally increased in *heg1^∆25^* mutant embryos compared with wt embryos. Besides, the expressions of vascular markers, such as *sox7*, *flk1*, *flt4*, *c-myb* [34] were also abnormally increased in *heg1^∆25^* mutant embryos (Figure 4E). This indicated cardiovascular malformations by loss of heg1 function in zebrafish.

In general, blood flow velocity of *heg1^∆25^* mutants was significantly lower than that of wt embryos. The venous congestion of *heg1^∆25^* mutants was very similar to that of zebrafish thrombosis model induced by physical and/or chemical treatments.

### 3.5. Application of heg1^∆25^ Mutants in Screening TCM Herbs for Cardiovascular Diseases Treatment

In order to verify the feasibility of *heg1^∆25^* mutants as a model of human heart failure and thrombosis, four known TCM herbs for the treatment of cardiovascular diseases or as a component of compound Chinese medicines for the treatment of cardiovascular diseases were selected to verify the model. *Radix astragali* (Huangqi) has been implicated in promoting endothelial cell proliferation and migration during angiogenesis [35,36]. The active ingredients of *Salviae miltiorrhiza* (Danshen) have antithrombotic and anticoagulant effects, and promote vascular remodeling and angiogenesis [37]. *Hirudo* (Shuizhi) can inhibit vascular smooth muscle cell (VSMC) proliferation, and the VSMC proliferation plays an important role in the formation of atherosclerotic plaque [38]. *Myrrh* (Moyao) is usually used as a component of compound Chinese medicines for the treatment of cardiovascular diseases, applied as an anticoagulant [39].

**Figure 5:**
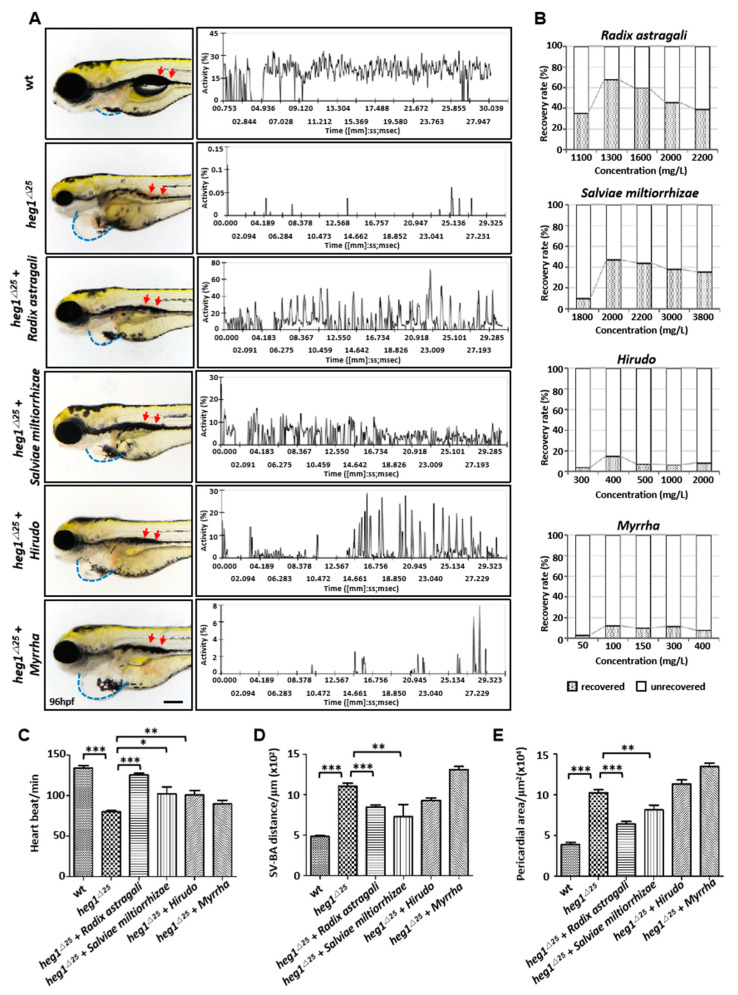
TCM pharmacological validation of *heg1^∆25^* zebrafish mutant. (**A**) Phenotype observation and the relative movement of RBCs in PVC analysis of *heg1^∆25^* embryos treated with four TCM herbs. (**B**) The optimal concentrations of the four drugs, as indicated as *Radix astragali* 1300 mg/L, *Salviae miltiorrhiza* 2000 mg/L, *Hirudo* 400 mg/L, and *Myrrha* 100 mg/L, respectively. N = 40 embryos for each concentrations. (**C**) Comparison of heart rate of zebrafish embryos in each group. (**D**) Comparison of SV-BA distance of zebrafish embryos in each group. (**E**) Comparison of pericardial area of zebrafish embryos in each group. Data are represented as mean ± SE from three independent experiments, * *p* < 0.05, ** *p* < 0.01, and *** *p* < 0.001 (Student’s *t*-test).

Zebrafish *heg1^∆25^* embryos at 48 hpf were treated with each drug to determine the optimal concentration for recovery. After two days of treatment, *heg1^∆25^* embryos treated with *Radix astragali* or *Salviae miltiorrhiza* exhibited significantly reduced venous congestion in PCV (red arrow) (Figure 5A), and the relative movement of RBCs in PCV were also obviously recovered (Figure 5A). Hirudo also showed a certain effect on venous thrombosis (red arrow), but had no obvious effect on the recovery of RBCs’ movements (Figure 5A). The optimal concentrations of the four drugs are shown in Figure 5B: *Radix astragali* 1300 mg/L, *Salviae miltiorrhiza* 2000 mg/L, *Hirudo* 400 mg/L, and *Myrrha* 100 mg/L, respectively. We next further analyzed the restoring phenotype of *heg1^∆25^* mutants at the optimal concentration of each drug. The heart rate of *heg1^∆25^* at 96 hpf was reduced to 80 ± 2.6 BPM compared with the heart rate of 130 ± 5.5 in wt group. Treatment with *Radix astragali* results in mutants’ heart rate returning to 121 ± 2 BPM. *Salviae miltiorrhiza* and *Hirudo* also showed different degrees of recovery to heart rate (Figure 5C). Since *heg1^∆25^* embryos had an enlarged atrium and ventricle, the SV-BA distance and the pericardial area were increased aberrantly in *heg1^∆25^* embryos. *Radix astragali* and *Salviae miltiorrhiza* treatment could reduce the abnormal SV-BA distance and significantly restore the pericardial edema of *heg1^∆25^* embryos (Figure 5A,D,E). Therefore, the cardiovascular phenotype of *heg1^∆25^* mutants was restored in different degrees under the treatment of *Radix astragali*, *Salviae miltiorrhiza*, and *Hirudo.*

### 3.6. Application of heg1^∆25^ mutants in Screening monomers for Cardiovascular Diseases Treatment

To further validate the reliability and diagnostic significance of the *heg1^∆25^* mutants in drug screening, we selected three bioactive components of TCM for the treatment of cardiovascular diseases to verify their rescue effect. Salvianolic acid B can promote angiogenesis and lesion plaque stability, and reduce acute myocardial infarction and atherosclerosis [40]. Paeoniflorin can inhibit acute myocardial infarction, ischemic stroke and atherosclerosis, and has a function of reducing acute myocardial infarction, ischemic stroke and atherosclerosis [41,42]. Ferulic Acid exhibits hypoglycemic and anti-oxidant effects, and has a certain therapeutic effect on diabetic cardiomyopathy (DCM) in rats [43,44]. Aspirin (acetylsalicylic acid, ASP) is one of the main therapeutic interventions for almost all patients with increased risk of thrombosis, and here it serves as a positive control.

**Figure 6:**
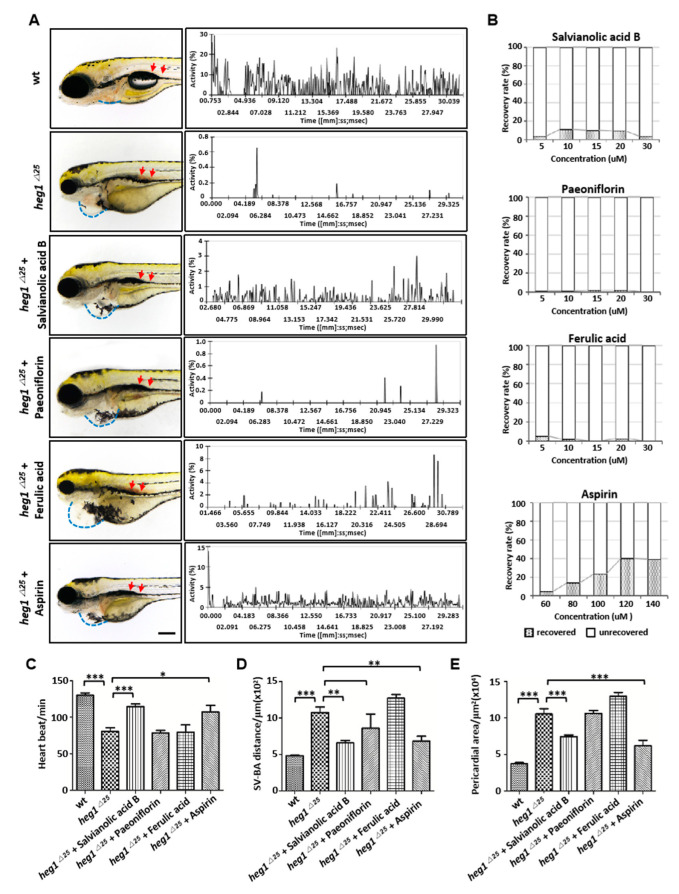
Monomers pharmacological validation of *heg1^∆25^* zebrafish mutants. (**A**) Phenotype observation and the relative movement of RBCs in PVC analysis of *heg1^∆25^* embryos treated with four monomers. (**B**) The optimal concentrations of the four monomers, salvianolic acid B, paeoniflorin, ferulic acid and aspirin. N = 40 embryos for each concentration. (**C**) Comparison of heart rate of zebrafish embryos in each group. (**D**) Comparison of SV-BA distance of zebrafish embryos in each group. (**E**) Comparison of pericardial area of zebrafish embryos in each group. Data are represented as mean ± SE from three independent experiments, * *p <* 0.05, ** *p* < 0.01, and *** *p* < 0.001 (Student’s *t*-test).

As shown in Figure 6, salvianolic acid B exhibited the best recovery ability to *heg1^∆25^* mutants in venous thrombosis (Figure 6A, red arrow), RBCs movements (Figure 6A), heart rate (Figure 6C), SV-BA distance (Figure 6D) and the pericardial area (Figure 6E) at the optimal concentration (Figure 6B), which was similar to that of aspirin. However, paeoniflorin and ferulic acid had no significant effect on the recovery of *heg1^∆25^* mutant cardiovascular phenotype.

## 4. Discussion

The *heg1^∆25^* zebrafish mutant described here is a typical model that recapitulates features of human cardiovascular diseases. We had shown that *heg1^∆25^* mutants exhibited DCM and venous thrombosis, including cardiac dilation, lower heart rate, PCV congestion, and reduced blood circulation. Additional molecular analysis of *heg1^∆25^* mutants showed that the expression levels of cardiovascular-development-related genes were abnormal. Further detailed analysis revealed that *heg1^+^^/^^∆25^* heterozygous embryos have no abnormal phenotype in comparison with wt embryos (Figure S2). In terms of the drug screening application. Furthermore, four TCM herbs (*Salviae miltiorrhiza*, *Radix astragali*, *Hirudo* and *Myrrha*) and three Chinese herbal monomers (salvianolic acid B, paeoniflorin, and ferulic acid) were used to treat *heg1^∆25^* mutants. We found that *Salviae miltiorrhiza*, *Radix astragali*, and salvianolic acid B could significantly restore the formation of thrombosis and relieve the heart failure in dilated cardiomyopathy of *heg1^∆25^* mutant. Our studies indicated that the *heg1^∆25^* mutant could be a valuable tool for the understanding and treatment of CVD.

In terms of those currently known, the therapeutic effects of TCM on CVDs are reflected by attenuating the damage of cardiomyocytes, endothelial cells (ECs), vascular smooth muscle cells (VSMCs) and macrophages/monocytes (M&Ms) [45]. The four TCM herbs and three monomers used in our study are commonly used medications for the treatment or prevention of CVD [45,46,47], while they have different targets and effects in vivo. Among the three monomers, salvianolic acid B exhibited the best dose-dependent recovery effect on *heg1^∆25^* mutant. Previous studies revealed that salvianolic acid B could inhibit the expression of vascular EGF protein in HUVEC cells by modulation of the ERK signaling pathway, as a result, to attenuate the disorganization of vascular endothelial-cadherin [47]. Salvianolic acid B also could inhibit TNFα-induced endothelial permeability [48]. Combined with the characteristics of the *heg1^∆25^* mutant, the enhanced adhesion of endothelial cells might be the reason for phenotype recovery in the treatment group. Salvianolic acid B is the extract of *Salviae miltiorrhiza*. Compared with salvianolic acid B, *Salviae miltiorrhiza* treatment group had a better curative effect. *Salviae miltiorrhiza* is widely used in the treatment of angina pectoris and acute ischemic stroke. Its effects mainly include dilation of coronary artery, improvement in microcirculation, inhibition of platelet adhesion and aggregation. Lin et al. reported that *Salviae miltiorrhiza* could reduce the phosphorylation of myosin light chain and facilitate the contraction of smooth muscle cells [45]. *Salviae miltiorrhiza* contains two types of major bioactive components, including water-soluble salvianolic acid and fat-soluble tanshinone. Rat acute myocardial infarction (MI) model experiment showed that salvianolic acid and tanshinone both could delay the development of ischemia by decreasing the infarct size and improving the systolic function after myocardial infarction [49]. Tanshinone acts in inhibition of intracellular calcium and cell adhesion. Salvianolic acid can mediate in oxidative stress genes downregulation, G-protein-coupled receptor activities and cell apoptosis in the late stage of ischemia [50]. The effect of various active components may be the reason why *Salviae miltiorrhiza* is more effective than salvianolic acid B.

In addition to *Salvia miltiorrhiza* and salvianolic acid B, *Radix astragali* also has a good effect on the treatment of *heg1^∆25^* mutant mutant thrombosis and heart failure in a dose-dependent manner. However, some monomers such as paeoniflorin, and ferulic acid had no significant dose-dependent effect compared with aspirin. As seen in our results, TCM herbs seems to have a better therapeutic effect than herbal monomers. This may be related to the characteristics of the “multi-component, multi-target, and multi-pathway” of TCM [51]. To date, many TCM have not been fully studied, and their mechanism in the prevention and treatment of cardiovascular diseases are still unclear [52]. Thus, our study further supports that TCM are valuable in the prevention and treatment of CVD.

The zebrafish system is becoming more and more popular in studying the molecular mechanism of human diseases and drug screening. However, as a lower vertebrate model system, zebrafish *heg1^∆25^* mutant is inevitably limited as a DCM or venous thrombosis model. Zebrafish heart has only one atrium and one ventricle; the anatomical structure of the cardiovascular system is much different from that of humans [4], so *heg1^∆25^* mutants cannot accurately reflect the phenotype of human cardiovascular diseases. Further work is needed to verify the efficacy and safety of the screened drugs in other vertebrate model systems, such as mice. Different from the single phenotype obtained by drug modeling (such as terfenadine-induced dilated cardiomyopathy model [53]), the *heg1^∆25^* mutant, as a congenital gene mutation model, has multiple developmental defects, which may bring some difficulties to specific drug screening and drug efficacy evaluation. However, as a gene knockout model, *heg1^∆25^* mutant maybe more sensitive to heg1-related signaling pathways and regulatory genes, rather than being used in general drug screening for a broad range of pathologies [19,20]. These aspects must be taken into consideration in future studies and applications of *heg1^∆25^* mutant. This will help us to further understand the regulatory pathways that drive these phenotypes, and even may even help us to screen relevant gene-targeted drugs.

Overall, zebrafish is an ideal model for screening and optimizing the timing and dosage of drugs because it can easily produce many embryos and allow the rapid evaluation of drug therapy effects. As a model of cardiovascular disease, *heg1^∆25^* mutant zebrafish has important advantages in the pharmacological research of CVD in the future, and will also contribute to the in-depth study on the pathogenesis of CVD.

## 5. Conclusions

CVD is one of the most frequent reasons for morbidity and mortality in the world. The *heg1^∆25^* zebrafish mutant we described here can be used as an effective model for the study of heart failure and venous thrombosis caused by human DCM. Some TCM herbs such as *Salviae miltiorrhiza*, *Radix astragali*, and monomers such as salvianolic acid B, have promising therapeutic effects on the heart failure and venous thrombosis of *heg1^∆25^* mutants. Taken together, the *heg1^∆25^* mutant strain has broad application prospects in drug screening and therapeutic treatment of CVD, and should be widely used in future.

## Supplementary Materials

The following are available online at https://www.mdpi.com/2218-273X/10/11/1542/s1, Table S1: List of all primer sequences. Figure S1: Analysis of *heg1^∆25^* mutant embryos. Figure S2: Comparison of wt, *heg1^+^^/^^∆25^* heterozygous and *heg1^∆25^* homozygous mutants.
