# Supplementary material for: Generation and Application of the Zebrafish heg1 Mutant as a Cardiovascular Disease Model
Source: Biomolecules. 2020 Nov 12;10(11):1542. doi: 10.3390/biom10111542 (PMC7696531; doi:10.3390/biom10111542)
Supplement: Supplementary file 1 [file biomolecules-10-01542-s001.pdf]

Supplementary materials

Gene Name	Forward 5'-3'	Reverse 5'-3'
heg1-exon1	GTGGAAACCGCTATCGCCTG	GTCGCAGGACCTAGTTGACTG
heg1-qPCR	CCACTGCCACAGCCGTGGATC	GTCAGATTGAAGATGTTCTG
β-actin	ATGGATGAGGAAATCGCTGCC	CTCCCTGATGTCTGGGTCGTC
myh6	GACATGGCGATGCTGACGTTTC	GATAAGCATTATCTGAGATG
ifabp	CTGAAGATCACCCTGGAGCAG	GTTGTCCTTGCGTGTGAAAG
cmlc2	GAGCTGGAGTCCATGCTAAC	CATTAGCAGCCTCTTGAACTCA
sox7	GGAGACCCATGAACGCCTT	GCTCGGCTTCCTCCACATAT
flk1	GACCATAAAACAAGTGAGGCAGAAG	CTCCTGGTTTGACAGAGCGATA
scl	GCCAATGGTGAAGTTGTGAGT	CGTCTGCTCTCTACCTGGAT
flt4	ATTACAACTGCGTGCCGTTT	TGTCAACATGGCTCCTCTGT
c-myb	TGAATCATCACGGGTGCCAT	TGTTGTCCCTTCAGCTCGTT
vegfba	ATCCTCCCTCCTGTGAATGC	CCTCAAAGTTGGATCGGTGG

Table S1. List of all primer sequences used for genotypes identify and Quantitative PCR.

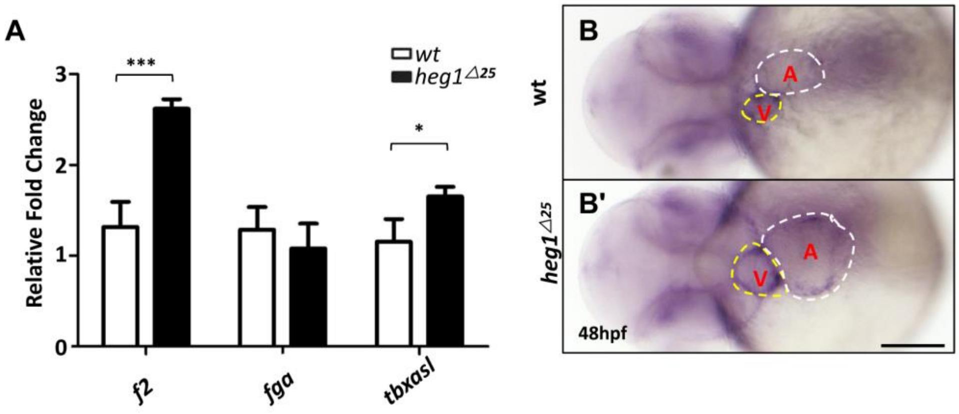

Figure S1. Analysis of *heg1*$^{\Delta 25}$ mutant embryos. (**A**) The expressions of thrombotic markers, as determined by qRT-PCR, were significantly changed in *heg1*$^{\Delta 25}$ mutants at 48 hpf. (**B**,**B'**) Representative images of the *heg1*$^{\Delta 25}$ and wt embryos at 48 hpf stained for the heart marker *bmp4*. Note the enlargement heart in *heg1*$^{\Delta 25}$ mutants (V: Ventricular, yellow dotted-line boxes; A: atria, white dotted-line boxes, ventral view). Data are represented as mean ± SE from three independent experiments, *$p < 0.05$, and ***$p < 0.001$ (Student's t-test).

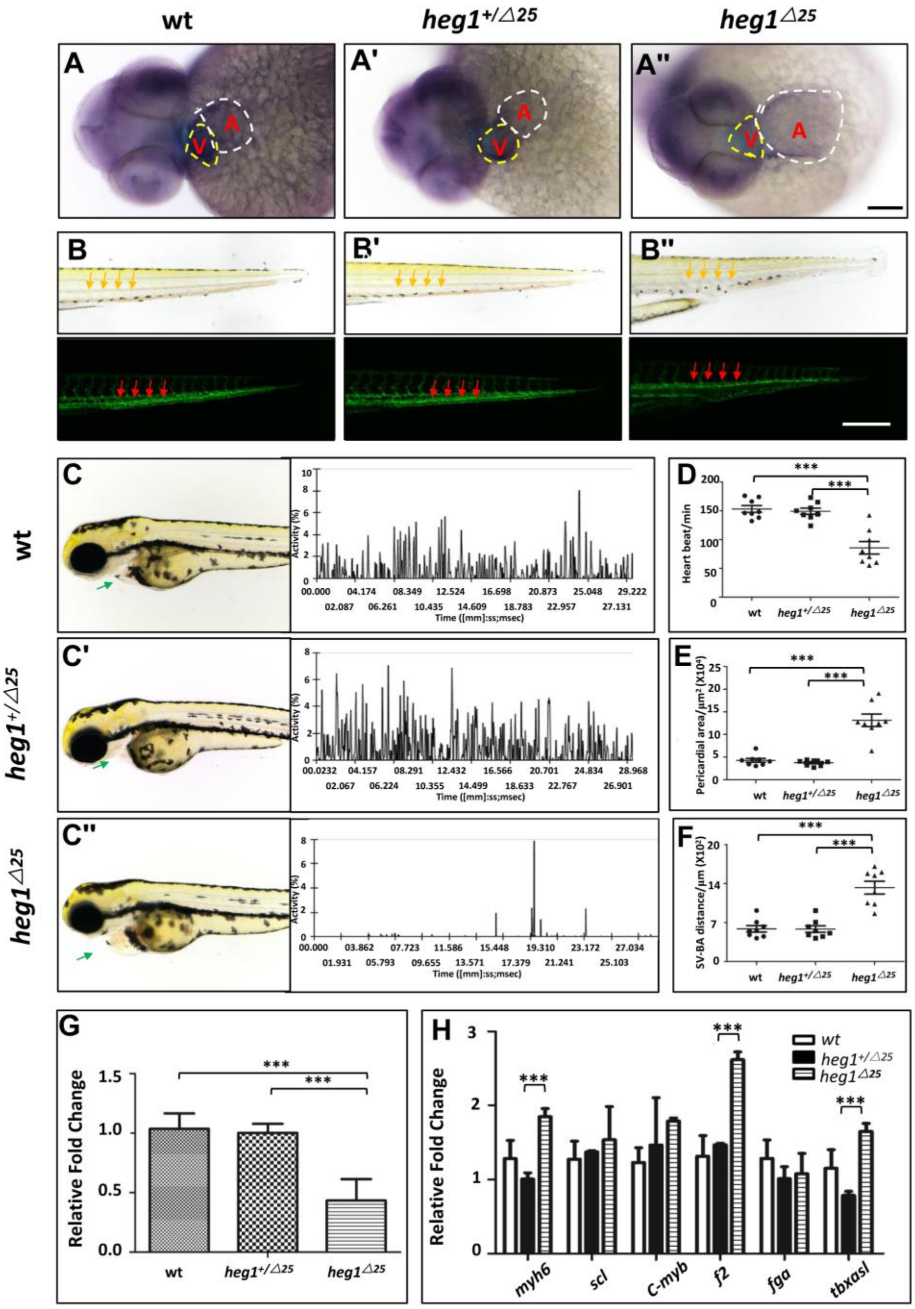

Figure S2. Comparison of wt, *heg1*$^{+/\Delta25}$ heterozygous and *heg1*$^{\Delta25}$ homozygous mutants. (**A,A',A''**) Representative images of the wt, *heg1*$^{+/\Delta25}$ heterozygous and *heg1*$^{\Delta25}$ homozygous mutant embryos at 48 hpf stained for the heart marker *cmlc1*, note the enlargement heart in *heg1*$^{\Delta25}$ mutants but not in wt and *heg1*$^{+/\Delta25}$ heterozygous embryos (V: Ventricular, yellow dotted-line boxes; A: atria, white dotted-line boxes, ventral view). (**B,B',B''**) Lateral view of zebrafish larvae at 96 hpf. Representative images of wt, *heg1*$^{+/\Delta25}$ heterozygous and *heg1*$^{\Delta25}$ homozygous mutants embryos, exhibiting blood congestion (yellow arrows), and dilation of dorsal aorta (DA) lumen (red arrows) in *heg1*$^{\Delta25}$ embryos but not in wt and *heg1*$^{+/\Delta25}$ heterozygous embryos. (**C,C',C''**) Lateral view of zebrafish larvae at 72 hpf. Representative images of wt, *heg1*$^{+/\Delta25}$, and *heg1*$^{\Delta25}$ embryos. The movement ratio of RBCs based on changes in pixel density of PVC. (**D**) Heart rate in wt, *heg1*$^{+/\Delta25}$, and *heg1*$^{\Delta25}$ zebrafish larvae (n=8 embryos/group). (**E**) The pericardial area in wt, *heg1*$^{+/\Delta25}$, and

heg1$^{\Delta 25}$ zebrafish larvae (n=8 embryos/group). (**F**) The SV-BA distance in wt, *heg1*$^{+/\Delta 25}$, and *heg1*$^{\Delta 25}$ zebrafish larvae (n=8 embryos /group). (**G**) qRT-PCR confirmation that *heg1* expression was significantly decreased in *heg1*$^{\Delta 25}$ embryos, no difference between wt and *heg1*$^{+/\Delta 25}$, n=30 embryos per group. (**H**) The expressions of cardiovascular markers, as determined by qRT-PCR at 48 hpf. Data are represented as mean ± SE from three independent experiments, ***$p < 0.001$ (Student's t-test).
